# E-cigarette use among Ecuadorian adults: A national cross-sectional study on use rates, perceptions, and associated factors

**DOI:** 10.18332/tid/187878

**Published:** 2024-05-31

**Authors:** Juan S. Izquierdo-Condoy, Patricio Naranjo-Lara, Estefanía Morales-Lapo, Alexander Puglla-Mendoza, Marlon R. Hidalgo, Andrea Tello-De-la-Torre, Eduardo Vásconez-González, Nathaly Izquierdo-Condoy, David Sánchez-Ordoñez, Galo S. Guerrero-Castillo, Raúl F. De la Rosa, Paul Vinueza-Moreano, Romina Placencia-André, M. Fernanda Díaz, Esteban Ortiz-Prado

**Affiliations:** 1 Facultad de Medicina, Universidad de las Américas, Quito, Ecuador

**Keywords:** e-cigarettes, perceptions, percentage of use, public health, control measures

## Abstract

**INTRODUCTION:**

Electronic cigarettes (e-cigarettes) have emerged as a new paradigm in nicotine delivery systems. Although they are marketed as safer alternatives to tobacco, public perceptions of their safety and utility vary widely. This study aims to understand the percentage of use, factors associated, perceptions, and attitudes about e-cigarettes among Ecuadorian adults.

**METHODS:**

A cross-sectional survey was conducted among the Ecuadorian population aged 18–65 years through a convenience sample, using a structured online questionnaire designed to collect responses from voluntary participants over three months, from February to April 2023. The questionnaire assessed the respondents’ attitudes and perceptions towards e-cigarettes. Data were analyzed using descriptive statistics, chi-squared tests, and adjusted logistic regression analyses to identify factors associated with e-cigarette use.

**RESULTS:**

Out of a total of 3047 Ecuadorian adults, the percentage of e-cigarette ever use was 27.9% (n=850), with 19.4% being current users and 8.5% former users. A negative stance towards e-cigarettes was predominant, with 66.3% considering e-cigarette use a public health problem in Ecuador. A significant association was observed between e-cigarette use and perceived harmfulness (p<0.001). Among non-users, there was a predominant stance in favor of control measures and disapproval of e-cigarette use among minors (p<0.001). The factors associated with the use of electronic cigarettes included being health personnel (AOR=1.51; 95% CI: 1.26–1.80). Older age (aged >24 years) and a history of tobacco use were associated with lower e-cigarette use (current users, OR=0.31; 95% CI: 0.25–0.38; previous users, OR=0.23; 95% CI: 0.18–0.28).

**CONCLUSIONS:**

The findings highlight a significant percentage of e-cigarette use among Ecuadorian adults, especially among younger groups. There is a need for comprehensive public health education about e-cigarettes in Ecuador. There is strong support from the public for control measures, suggesting the potential acceptability of regulations concerning e-cigarettes.

## INTRODUCTION

Tobacco use has consistently been at the forefront of global health challenges, causing significant mortality and morbidity^1,2^. According to the World Health Organization (WHO), around 1.3 billion people globally use tobacco, leading to 8 million deaths annually from its consumption^3^. To combat its detrimental effects, numerous countries have introduced public policies, such as restrictions on smoking in public areas and tobacco taxation^4^. Such strategies have been especially pronounced in developed countries with high standards of regulatory control^5^.

Emerging as alternatives to conventional tobacco products, Electronic Nicotine Delivery Systems (ENDS) or electronic cigarettes, were introduced in 2006. These devices have witnessed rapid adoption, particularly among the younger generation, with approximately 68 million individuals worldwide estimated to use e-cigarettes as of 2021^6,7^. Nowadays, the e-cigarette market features hundreds of brands, commonly using cartridges filled with glycerin or propylene glycol and varying nicotine levels^8^. While the full risks of these battery-operated devices remain unclear, they are often viewed as a safer alternative to traditional cigarettes and a potential tool for quitting smoking^9^. Research indicates strong positive perceptions about e-cigarettes, with a study highlighting 45% of participants seeing them as a cessation aid^10^.

Perceptions surrounding e-cigarettes vary. A study involving British youths found that 63.4% considered e-cigarettes less harmful than their traditional counterparts. In contrast, only 34.2% of American youths shared this sentiment^11,12^. Among adults, a meta-analysis comprising 28 studies indicated that 61.2% view e-cigarettes as a more health-conscious alternative^13^.

In Latin America, due to the sustained efforts under the WHO Framework Convention for Tobacco Control (FCTC), there has been a noticeable decline in tobacco consumption from 28% in 2000 to 16.3% in 2020^14,15^. In Ecuador, a survey conducted in 2016 reported that among students aged 13–15 years, the prevalence of tobacco consumption stood during the last 15 years at 13%^16^. However, the ENDS industry’s landscape in the region has shifted considerably. By 2020, its retail value had risen from $21 million in 2015 to $94.2 million^17^, signaling an increased adoption among younger individuals who had not previously smoked^15^.

Despite the increasing popularity of e-cigarettes, especially in developing regions like Latin America, comprehensive studies into their consumption patterns remain sparse. Ecuador needs more detailed research to understand e-cigarette-related perceptions and beliefs in its adult population. Addressing this gap, our study seeks to estimate factors associated with e-cigarette use and assess the underlying perceptions within Ecuador’s adult demographic.

## METHODS

### Study design and setting

This study was a nationwide descriptive cross-sectional survey carried out in Ecuador over a span of three months, from February to April 2023. The research utilized a self-administered online questionnaire.

Ecuador, a Latin American country located along the equatorial line, is nestled within the Andean mountainous region and bordered by the Pacific Ocean. Sharing its borders with Peru and Colombia, this small nation had a population of approximately 17.6 million inhabitants as of 2021^18^. In Ecuador, no specific laws have been created focused on electronic cigarettes. Thus, the regulation and control of the use of electronic cigarettes have been established theoretically by including them within the framework of the Organic Law on Tobacco Regulation and Control^19^.

### Population and study size

The study participants comprised adult residents of Ecuador, defined as individuals aged 18–65 years. As of 2021, the reported adult population (aged 18–65 years) in Ecuador was approximately 10 million^20^. Given a 95% confidence level and a margin of error set at 2%, the study required a minimum sample size of 2401 respondents. The required sample size was estimated using standard formula for sample size calculation in population surveys^21^.

A non-probability convenience sampling method was employed to select participants within the researchers’ reach using an online survey platform. All responses included were obtained from voluntary participants who agreed to take part in the research.

### Survey development and measures

The study employed a 32-item anonymous online questionnaire designed by the research team to collect data on the percentage of use, perceptions, and beliefs about electronic cigarette use in Ecuador’s adult population.

The initial version of the questionnaire underwent review by two public health experts for relevance, accuracy, and potential errors. Following feedback, the questionnaire was pilot-tested with 30 eligible participants to identify any comprehension or design issues. Responses from this pilot phase were not included in the final survey data.

After the pilot test, revisions were made to produce the final version of the questionnaire, which was originally drafted in Spanish and later translated into English for this study. The final survey instrument is included in the Supplementary file.

### Variables

The questionnaire collected various variables to address the research objective. Demographic variables included sex, age, race, geographical region of residence, marital status, education level, type of professional activities (health-related, non-health-related), and a history of comorbidities.

Additionally, the history of tobacco use (non-use, past use, current use) and e-cigarette use (non-use, past use, current use) were assessed. However, to identify factors associated with e-cigarette use, these variables were recategorized into two groups (use and non-use).

Furthermore, several variables were evaluated regarding the beliefs and perceptions of the population about electronic cigarettes, including beliefs about electronic cigarette components (nicotine, tobacco), characteristics of electronic cigarette use (capacity to generate addiction, health effects, effects on others), public health effects (use by minors, a tool to quit smoking, restrictions on use, taxes), and characteristics of the environment (how the electronic cigarette was known, friends and relatives who use EC, use of EC by parents).

### Data collection and management

**Figure 1:**
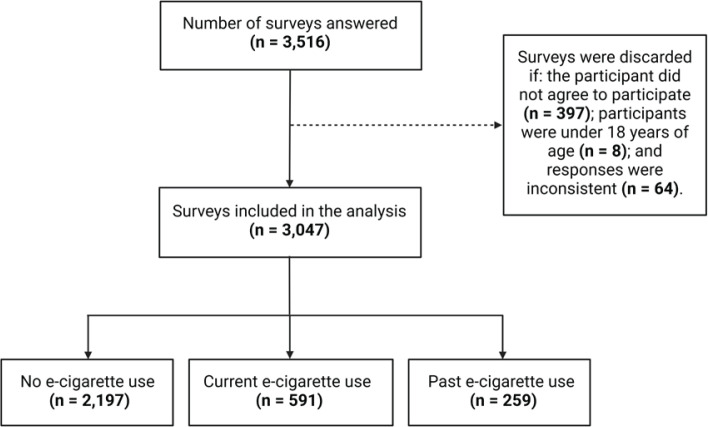
Flow chart of the survey participant selection process

The online platform ‘Survey Monkey’ facilitated data collection. Participants accessed the survey via a unique link disseminated across social media channels, including Facebook, WhatsApp, and Twitter. The preamble to the survey briefly outlined the study’s aims, promised confidentiality, and sought informed consent. Participants had to agree to the Terms and Conditions and accept the Participation Agreement to access the survey, reinforcing anonymity. To maintain high data integrity, we meticulously scrutinized all responses for potential errors or inconsistencies, such as implausible age ranges or respondents choosing all available answers, excluding these questionnaires from the final sample. Furthermore, entries with only demographic data filled were also discarded. Initially, we had 3516 survey responses. Post rigorous filtering, a total of 3047 responses were deemed valid and included in the study (Figure 1).

To control potential bias during data collection and management, we implemented several strategies. To prevent duplicate submissions, the ‘Survey Monkey’ platform was configured to limit one response per IP device. Additionally, the design of our questionnaire was intentionally curated to prevent the collection of identifiable information, such as IP addresses. To further minimize bias during the analysis phase, each member of the research team independently reviewed the results. When discrepancies surfaced, they were collaboratively resolved to ensure only valid and genuine responses were incorporated into our final analysis.

### Ethics

This study strictly adhered to the ethics standards outlined in the Declaration of Helsinki. Furthermore, it complied with the ethics protocols approved by the Ethics Committee of the Universidad de las Américas (CEISH-UDLA). Throughout the conduct of this study, we maintained the principles of participant anonymity and voluntary participation. The research neither solicited nor included any personally identifiable or sensitive information.

### Statistical analysis

Our study primarily utilized descriptive statistics to analyze the responses for each categorical item in the questionnaire, including the calculation of frequencies and percentages. To assess the differences between categorical variables, we employed the chi-squared test.

A logistic regression model was utilized to estimate the association between participant characteristics and EC use, expressed by odds ratio (OR) and 95% confidence intervals (95% CI). For this model, the group of current and past e-cigarette users was combined to obtain two categories: no EC use and EC use. Additionally, an adjusted multivariate analysis was performed for the variable ‘tobacco use’ (combining the group of current and past tobacco users to obtain two categories: non-tobacco use and tobacco use) as a potential confounder of the association between participants’ characteristics and EC use. The results of this analysis were expressed as adjusted odds ratio (AOR) and 95% CI.

Tests were two-tailed, and we deemed statistical significance at p<0.05. For both our descriptive and inferential analyses, we utilized the IBM SPSS Statistics software for Windows, version 24.0 (IBM Company, Chicago, IL, USA).

## RESULTS

### General characteristics

From a total of 3047 participants, the sample primarily consisted of females (54.6%), individuals of Mestizo race (93.1%), unmarried (69.0%), and those holding a university degree (34.4%). Most respondents (84.2%) reported no comorbidities. However, obesity (3.6%) and hypothyroidism (3.5%) were the most prevalent comorbidities. Among the respondents, 1090 (35.8%) identified as ever users of tobacco; of these, 53.9% are currently smoking, and 46.1% are former smokers. Most of these smokers reported a habit of fewer than five years and typically smoked fewer than ten cigarettes per day (Table 1).

**Table 1:** Demographic characteristics and history of e-cigarette use of Ecuadorian adult population, 2023 (N=3047)

*Characteristics*	*n*	*%*
**Sex**		
Male	1373	45.1
Female	1663	54.6
Prefer not to mention	11	0.4
**Age** (years)		
18–24	1048	35.2
25–34	1177	39.5
35–44	425	14.3
45–54	231	7.8
55–65	78	2.6
>65	18	0.6
**Race**		
Mestizo	2837	93.1
Indigenous	40	1.3
White	85	2.8
Afro-descendant	31	1.0
Montubio	42	1.4
Other	12	0.4
**Region of residence**		
Sierra	2571	84.4
Costa	409	13.4
Amazon	67	2.2
**Marital status**		
Single	2103	69.0
Married	638	20.9
Common-law marriage	113	3.7
Divorced	179	5.9
Widowed	14	0.5
**Education level**		
Incomplete school	5	0.2
School completed	27	0.9
Incomplete high school	72	2.4
High school completed	360	11.8
Incomplete university	1010	33.1
University degree	1048	34.4
Incomplete Master’s/PhD	178	5.8
Master’s/PhD degree	347	11.4
**Professional activities related to health**		
Yes	1477	48.5
No	1570	51.5
**Comorbidities**		
Diabetes mellitus type 1	14	0.5
Diabetes mellitus type 2	28	0.9
Asthma	70	2.3
Cancer	21	0.7
Hyperthyroidism	20	0.7
Hypothyroidism	109	3.6
Obesity	107	3.5
High blood pressure	82	2.7
Human immunodeficiency virus	5	0.2
Coagulation disorders	12	0.4
Chronic obstructive pulmonary disease	12	0.4
None	2567	84.2
**Tobacco use**		
No	1957	64.2
Current use	588	19.3
Past use	502	16.5
**Years of tobacco use**		
0–5	653	61.3
6–10	234	22.0
11–20	124	11.6
>20	54	5.1
**Cigarettes per day**		
1–10	836	84.1
11–20	96	9.7
21–40	40	4.0
>40	22	2.2
**Electronic cigarette use**		
No	2197	72.1
Current use	591	19.4
Past use	259	8.5

### Percentage of use and association of e-cigarette use

Of the total participants surveyed (n=3047), 27.9% (n=850) reported e-cigarette ever use, with 19.4% (n=591) identified as current users and 8.5% (n=259) as former users (Table 1). The data highlighted a clear disparity between tobacco ever users 35.8% (16.5% of former users and 19.5% of current users) and non-users 64.2%. Predominantly, male respondents accounted for a significant proportion of current or former e-cigarette users (p<0.001).

The percentage of use of e-cigarettes tended to be higher among younger respondents, with the percentage of current and past users diminishing progressively with increasing age (p<0.001) (Table 2). Furthermore, the analysis revealed that individuals engaged in health services, those without any existing comorbidities, and current tobacco smokers were more inclined towards e-cigarette use (p<0.05) (Table 2).

**Table 2:** Prevalence of electronic cigarette use according to the characteristics of Ecuadorian adult population, 2023 (N=3047)

*Characteristics*	*Total*	*EC use*						
		*No*		*Past use*		*Current use*		p*
	*n*	*n*	*%*	*n*	*%*	*n*	*%*	
**Sex**								**<0.001**
Male	1373	906	66.0	155	11.3	312	22.7	
Female	1663	1284	77.2	104	6.3	275	16.5	
Prefer not to mention	11	7	63.6	0	0.0	4	36.4	
**Age** (years)								**<0.001**
18–24	1048	673	64.7	104	10.0	263	25.3	
25–34	1177	794	68.1	111	9.5	261	22.4	
35–44	425	346	81.6	27	6.4	51	12.0	
45–54	231	215	93.5	6	2.6	9	3.9	
55–65	78	73	93.6	4	5.1	1	1.3	
>65	18	16	88.9	1	5.6	1	5.6	
**Race**								0.447
Mestizo	2837	2048	72.2	247	8.7	542	19.1	
Indigenous	40	30	75.0	2	5.0	8	20.0	
White	85	54	63.5	8	9.4	23	27.1	
Afro-descendant	31	24	77.4	1	3.2	6	19.4	
Montubio	42	33	78.6	1	2.4	8	19.0	
Other	12	8	66.7	0	0.0	4	33.3	
**Region of residence**								
Sierra	409	318	77.7	19	4.7	72	17.6	
Costa	2571	1828	71.1	231	9.0	512	19.9	
Amazon	67	51	76.1	9	13.4	7	10.5	
**Marital status**								**<0.001**
Single	2103	1423	67.7	202	9.6	478	22.7	
Married	638	541	84.8	35	5.5	62	9.7	
Common-law marriage	113	83	73.5	13	11.5	17	15.0	
Divorced	179	141	78.8	9	5.0	29	16.2	
Widowed	14	9	64.3	0	0.0	5	35.7	
**Education level**								**0.003**
Incomplete school	5	4	80.0	0	0.0	1	20.0	
School completed	27	25	92.6	0	0.0	2	7.4	
Incomplete high school	72	54	75.0	9	12.5	9	12.5	
High school completed	360	287	79.7	21	5.8	52	14.4	
Incomplete university	1010	689	68.2	94	9.3	227	22.5	
University degree	1048	753	71.9	97	9.3	198	18.9	
Incomplete Master’s/PhD	178	127	71.3	10	5.6	41	23.0	
Master’s/PhD degree	347	258	74.4	28	8.1	61	17.6	
**Professional activities related to health**								**0.002**
Yes	1477	1022	69.2	138	9.3	317	21.5	
No	1570	1175	74.8	121	7.7	274	17.5	
**Comorbidities**								**0.001**
Diabetes mellitus type 1	14	13	92.9	0	0.0	1	7.1	
Diabetes mellitus type 2	28	23	82.1	1	3.6	4	14.3	
Asthma	70	50	71.4	4	5.7	16	22.9	
Cancer	21	18	85.7	0	0.0	3	14.3	
Hyperthyroidism	20	17	85.0	2	10.0	1	5.0	
Hypothyroidism	109	88	80.7	6	5.5	15	13.8	
Obesity	107	74	69.2	8	7.5	25	23.4	
High blood pressure	82	74	90.2	2	2.4	6	7.3	
Human immunodeficiency virus	5	1	20.0	0	0.0	4	80.0	
Coagulation disorders	12	10	83.3	1	8.3	1	8.3	
Chronic obstructive pulmonary disease	12	11	91.7	1	8.3	0	0.0	
None	2567	1818	70.8	234	9.1	515	20.1	
**Tobacco use**								**<0.001**
No	588	265	45.1	63	10.7	260	44.2	
Current use	502	247	49.2	106	21.1	149	29.7	
Past use	1957	1685	86.1	90	4.6	182	9.3	
**Years of tobacco use**								0.183
0–5	653	298	45.6	103	15.8	252	38.6	
6–10	234	100	42.7	35	15.0	99	42.3	
11–20	124	55	44.4	21	16.9	48	38.7	
>20	54	34	63.0	8	14.8	12	22.2	
**Cigarettes per day**								**0.030**
1–10	836	383	45.8	129	15.4	324	38.8	
11–20	96	36	37.5	21	21.9	39	40.6	
21–40	40	11	27.5	9	22.5	20	50.0	
>40	22	5	22.7	3	13.6	14	63.6	

COPD: chronic obstructive pulmonary disease. EC: electronic cigarette. HIV: human immunodeficiency virus.

* p was calculated from the chi-squared test.

Regression analysis revealed that the younger age group (aged 18–24 years) had a stronger association with e-cigarette use than the age groups of 25–34 years (AOR=0. 69; 95% CI: 0.57–0.85), 35–44 years (AOR=0.29; 95% CI: 0.21–0.39), 45–54 years (AOR=0.09; 95% CI: 0.05–0.16), 55–65 years (AOR=0.07; 95% CI: 0.03–0.21) and >65 years (AOR=0.14; 95% CI: 0.02–0.92). On the other hand, married and divorced respondents showed a lower association towards using e-cigarettes compared to single participants (AOR=0.30; 95% CI: 0.23–0.39 and AOR=0.43; 95% CI: 0.29–0.65; respectively) (Table 3). The presence of comorbidities significantly affected e-cigarette use, as these individuals showed a lower association (AOR=0.60; 95% CI: 0.47–0.78) compared to those who did not report comorbidities. Meanwhile, performing health-related professional activities was a trait associated with e-cigarette use (AOR=1.51; 95% CI: 1.26–1.80). In addition, current (OR=0.31; 95% CI: 0.25–0.38) and former tobacco users (OR=0.23; 95% CI: 0.18–0.28) showed a lower association with e-cigarette use compared to non-smokers (Table 3).

**Table 3:** Factors associated with electronic cigarette use among Ecuadorian adult population, 2023 (N=3047)

*Variables*	*EC use*					
	*No n*	*Yes n*	*OR (95% CI)*	*p*	*AOR (95% CI)*	*p*
**Sex**						
Female ®	1284	379	1		1	
Prefer not to mention	7	4	1.94 (0.56–6.65)	0.294	1.08 (0.28–4.08)	0.914
Male	906	467	1.75 (1.49–2.05)	**<0.001**	1.18 (0.99–1.41)	0.065
**Age** (years)						
18–24 ®	673	367	1		1	
25–34	794	372	0.86 (0.72–1.03)	0.092	0.69 (0.57–0.85)	**<0.001**
35–44	346	78	0.41 (0.31–0.55)	**<0.001**	0.29 (0.21–0.39)	**<0.001**
45–54	215	15	0.13 (0.07–0.22)	**<0.001**	0.09 (0.05–0.16)	**<0.001**
55–65	73	5	0.13 (0.05–0.31)	**<0.001**	0.07 (0.03–0.21)	**<0.001**
>65	16	2	0.23 (0.05–1.00)	0.050	0.14 (0.02–0.92)	**0.040**
**Region of residence**						
Sierra ®	1828	743	1		1	
Coast	318	91	0.70 (0.55–0.90)	**0.005**	0.73 (0.55–0.96)	**0.026**
Amazon	51	16	0.77 (0.44–1.36)	0.371	0.84 (0.46–1.53)	0.563
**Race**						
Mestizo ®	2048	789	1		1	
Indigenous	30	10	0.87 (0.42–1.78)	0.693	1.09 (0.50–2.37)	0.823
White	54	31	1.49 (0.95–2.34)	0.081	1.54 (0.93–2.57)	0.094
Afro-descendant	24	7	0.76 (0.32–1.76)	0.645	1.09 (0.43–2.80)	0.857
Montubio	33	9	0.71 (0.34–1.49)	0.361	0.67 (0.29–1.59)	0.366
Other	8	4	1.30 (0.39–4.32)	0.425	0.86 (0.24–3.04)	0.814
**Marital status**						
Single ®	1423	680	1		1	
Married	541	97	0.38 (0.29–0.47)	**<0.001**	0.30 (0.23–0.39)	**<0.001**
Common-law marriage	83	30	0.76 (0.49–1.16)	0.200	0.69 (0.43–1.09)	0.112
Divorced	141	38	0.56 (0.39–0.82)	**0.002**	0.43 (0.29–0.65)	**<0.001**
Widowed	9	5	1.16 (0.39–3.48)	0.787	1.45 (0.42–5.04)	0.561
**Education level**						
High school completed ®	287	73	1		1	
Incomplete school	4	1	0.98 (0.11–8.93)	0.987	1.35 (0.10–17.91)	0.819
School completed	25	2	0.31 (0.07–1.36)	0.121	0.31 (0.06–1.47)	0.139
Incomplete high school	54	18	1.31 (0.72–2.37)	0.370	1.54 (0.82–2.88)	0.179
Incomplete University	689	321	1.83 (1.37–2.45)	**<0.001**	1.95 (1.43–2.66)	**<0.001**
University degree	753	295	1.54 (1.15–2.06)	**0.003**	1.51 (1.10–2.05)	**0.010**
Incomplete Master’s/PhD	127	51	1.58 (1.04–2.39)	**0.030**	1.55 (0.99–2.41)	0.052
Master’s/PhD degree	258	89	1.36 (0.95–1.93)	0.090	1.37 (0.93–2.00)	0.113
**Professional activities related to health**						
No ®	1175	395	1		1	
Yes	1022	455	1.32 (1.13–1.55)	**<0.001**	1.51 (1.26–1.80)	**<0.001**
**Comorbidities**						
None ®	1818	749	1		1	
Yes	379	101	0.65 (0.51–0.82)	**<0.001**	0.60 (0.47–0.78)	**<0.001**
**Tobacco use**						
No ®	1685	272	1			
Past use	247	255	0.23 (0.18–0.28)	**<0.001**		
Current use	265	323	0.31 (0.25–0.38)	**<0.001**		

AOR: adjusted odds ratio. EC: electronic cigarette. ® Reference categories.

### General perceptions of electronic cigarettes

Our study revealed a divided understanding among Ecuadorians about certain aspects of electronic cigarettes (e-cigarettes), such as their nicotine or tobacco content. This aligns with the participants’ self-assessment, with 44.0% (n=1250) acknowledging minimal knowledge and 20.0% (n=539) reporting no knowledge at all about e-cigarettes (Supplementary file).

Negative attitudes towards e-cigarettes were predominant. A notable portion of participants believed e-cigarettes offer no health benefits (43.3%, n=1232), and their harmful effects are equivalent to those from tobacco use (53.9%, n=1534). This negativity is reflected in the belief that e-cigarette use constitutes a current public health issue in Ecuador, as agreed by 66.3% (n=1885) of participants (Supplementary file).

Important trends were reported, with 73.7% (n=2096) of participants witnessing minors using e-cigarettes. Many believe this may lead to an earlier start in tobacco use among new users (73.8%, n=2100) or prompt former smokers to resume smoking (68.7%, n=1953). As a result, most participants advocated for control measures on e-cigarette use, such as implementing additional taxes (70.1%, n=1962) and restrictions in public areas (67.6%, n=1922) (Supplementary file).

### Perceptions according to e-cigarette use

The analysis indicated a relationship between the use of e-cigarettes and perceptions of them. Non-users of e-cigarettes were more inclined to categorize them as very harmful in 83.3% (p<0.001). In contrast, individuals who currently use e-cigarettes had a stronger tendency to perceive them as less harmful than traditional tobacco in 25.2% (p<0.001). Moreover, the advocacy for control measures, such as the imposition of additional taxes and disapproval towards underage e-cigarette use, were predominantly seen among non-e-cigarette users in 83.9% (p<0.001) and 75.4% (p<0.001), respectively. Notably, the environmental influence was evident in our findings; most respondents (49.5%) who reported their parents as e-cigarette users were also users themselves (p<0.001) (Supplementary file).

Supplementary file Figure 1 depicts the main characteristics of ever users of electronic cigarettes and Supplementary file Figure 2 summarizes the most important factors associated with the use of electronic cigarettes.

## DISCUSSION

Our study, conducted through an online questionnaire, identified a significant prevalence of e-cigarette use among Ecuadorian adults, with 27.9% reporting ever use and 19.4% as current users. These rates are higher than recent estimates in the adult populations of Nigeria and Ireland ^22,23^. Within the e-cigarette user group, the highest number of participants was concentrated among individuals aged 18–34 years, male, single, health professionals, and tobacco users (Figure 1).

Regarding the factors associated with the use of electronic cigarettes, although consistent with previous studies^24,25^, e-cigarette use was more frequent among Ecuadorian adult men; however, male sex was not shown to be a factor associated with EC use. Additionally, the association of EC use decreased with age, with the youngest group (aged 18–24 years) being the most affected; this factor has been reported in recent years in several countries^23,26,27^. Likewise, it was found that having an education level of incomplete or complete university is associated with the use of electronic cigarettes; this could be related mainly to the fact that the participants of these groups are in the younger age ranges.

On the other hand, performing professional activities in the health area was shown to be a factor associated with e-cigarette use, although other research has shown that a significant percentage of healthcare workers use tobacco^28^, and this could be related to e-cigarette use. We believe that the estimate was influenced because about 50% of the sample was made up of healthcare workers, a trait that does not represent the Ecuadorian population.

Meanwhile, although Aljandaleh et al.^29^ have found that having comorbidities such as obesity is associated with e-cigarette use, in our population, the effect was the opposite, as having comorbidities was associated with less e-cigarette use. Interestingly, we found that smoking tobacco (currently or in the past) was associated with less e-cigarette use. This finding is particularly interesting given that several previous investigations have found strong associations between smoking history and e-cigarette use^22,23,27,29^. We believe that this discrepancy may demonstrate that e-cigarette use as a smoking cessation tool is being relegated and is beginning to migrate to non-user groups.

Regarding participants’ perceptions, a surprising revelation from our study was that, despite the significant number of e-cigarette users, the majority of participants, regardless of whether they used e-cigarettes or not, held negative perceptions about them. This includes not perceiving health benefits and considering that electronic cigarettes bring harmful effects like those of tobacco. Similarly, Malt et al.^30^ reported an increase in negative perceptions toward electronic cigarettes in US adults. Despite the evidence regarding the harmful effects of e-cigarettes, their global popularity, particularly among the youth, continues to surge^31^. There exists a significant knowledge gap about their understanding and usage, especially in developing countries^31^. In Ecuador, the Organic Law for the Regulation and Control of Tobacco mandates regulations on e-cigarettes. It forbids selling to and using them by those aged <18 years and bans use in public areas^19^. However, our study discovered that 73.7% of participants observed minors using e-cigarettes, spotlighting regulatory shortcomings. This finding is particularly important given that most e-cigarettes contain nicotine, which can lead to addiction, especially in adolescents, and can act as a gateway to other drugs^10^. Research indicates that e-cigarette trials might increase the risks of adopting tobacco and marijuana habits, contradicting the tobacco decline since the 1970s^32,33^. Additionally, a significant 74% of our participants believe e-cigarettes ease the transition to tobacco smoking.

The e-cigarette industry has promoted its products as healthier alternatives to smoking. Although studies have supported these claims, it has been revealed that research has been supported by e-cigarette producers^34,35^. It is likely that this misinformation, combined with other influences, is accelerating the consumption of e-cigarettes worldwide, especially among vulnerable groups such as minors, who may perceive them as benign or normal^36^.

Ecuador faces regulatory challenges, especially given the easy accessibility of e-cigarettes in convenience stores, vending machines, etc.^37^. This aligns with the majority of participants in our study citing personal acquaintances and social networks for their primary introduction to e-cigarettes, which is consistent with findings from other countries such as Jordan and Egypt^10,31^. The role of the tobacco industry in this, with its attractive product designs and extensive social media campaigns, cannot be overlooked^38^.

Despite the consensus in favor of strict regulation of e-cigarettes like that of traditional cigarettes, there is a significant gap in Ecuador in terms of awareness and enforcement. This study paves the way for further research on e-cigarette consumption habits, product components in Ecuador, and associated health effects in developing countries. The influence of the tobacco industry, especially its uncontrolled sale in e-cigarette vending machines, deserves to be analyzed in future research.

### Limitations

Despite its valuable findings, our study has some limitations that should be acknowledged. Firstly, our sample was obtained through a self-reported online questionnaire, which might have introduced selection bias and potentially inflated the percentage of use rates. Moreover, our sampling strategy might have included a disproportionate number of individuals with access to social networks, which is not representative of the entire Ecuadorian population. In the same context, possibly because of the convenience sampling used, within the population of this research, a large percentage of participants were found to be tobacco users and to have professions related to the health sector. It is most likely that these traits are not representative of the entire Ecuadorian population. Also, the cross-sectional design of the study prevents us from making causal inferences and risk estimations. The lack of data on the type and frequency of e-cigarette use and concurrent use with conventional tobacco products, limits our understanding of usage patterns.

## CONCLUSIONS

Our study unveils a high percentage of electronic cigarette use among our sample of Ecuadorian adults. Young individuals and those without a history of smoking were found to be associated with e-cigarette use. These findings, coupled with negative perceptions towards e-cigarette use, emphasize the urgency for robust public health measures. There is a distinct call for comprehensive regulation to govern access to and use of e-cigarettes, especially among minors, and to rectify misconceptions about the safety of e-cigarettes in Ecuador.

## Supplementary Material



## Data Availability

The data supporting this research are available from the authors on reasonable request.

## References

[1] Patanavanich R, Glantz SA. Smoking is associated with worse outcomes of COVID-19 particularly among younger adults: a systematic review and meta-analysis. BMC Public Health. 2021;21:1-9. doi:10.1186/s12889-021-11579-x 34399729 PMC8366155

[2] Parascandola M, Xiao L. Tobacco and the lung cancer epidemic in China. Transl Lung Cancer Res. 2019;8(Suppl 1):S21. doi:10.21037/tlcr.2019.03.12 31211103 PMC6546632

[3] World Health Organization. Tobacco. July 31, 2023. Accessed January 27, 2024. https://www.who.int/news-room/fact-sheets/detail/tobacco

[4] Galanti MR, Coppo A, Jonsson E, Bremberg S, Faggiano F. Anti-tobacco policy in schools: upcoming preventive strategy or prevention myth? A review of 31 studies. Tob Control. 2014;23(4):295-301. doi:10.1136/tobaccocontrol-2012-050846 23716172

[5] World Health Organization. WHO Report on the Global Tobacco Epidemic, 2017: Monitoring Tobacco Use and Prevention Policies. World Health Organization; 2017. Accessed January 27, 2024. https://iris.who.int/bitstream/handle/10665/255874/9789241512824-eng.pdf?sequence=1

[6] Jerzyński T, Stimson GV, Shapiro H, Król G. Estimation of the global number of e-cigarette users in 2020. Harm Reduct J. 2021;18(1):109. doi:10.1186/s12954-021-00556-7 34688284 PMC8541798

[7] Feeney S, Rossetti V, Terrien J. E-cigarettes-a review of the evidence-harm versus harm reduction. Tob Use Insights. 2022;15:1179173X221087524. doi:10.1177/1179173X221087524 PMC8968985 35370428

[8] Caponnetto P. Well-being and harm reduction, the consolidated reality of electronic cigarettes ten years later from this emerging phenomenon: a narrative review. Health Psychol Res. 2021;8(3):9463. doi:10.4081/hpr.2020.9463 33553795 PMC7859958

[9] Zhao D, Abdullah AS, Wen T, et al. Perceptions of e-cigarettes among smokers and non-smokers in households with children in rural China: a cross-sectional study. Tob Induc Dis. 2021;19(April):25. doi:10.18332/tid/133264 33850512 PMC8033598

[10] Barakat M, Assaf AM, Al-Qudah R, et al. Perception of adults toward electronic cigarettes: a cross-sectional study from Jordan. Prim Health Care Res Dev. 2021;22:e3. doi:10.1017/S1463423621000062 33504409 PMC8057464

[11] East K, Brose LS, McNeill A, Cheeseman H, Arnott D, Hitchman SC. Harm perceptions of electronic cigarettes and nicotine: a nationally representative cross-sectional survey of young people in Great Britain. Drug Alcohol Depend. 2018;192:257-263. doi:10.1016/j.drugalcdep.2018.08.016 30300799 PMC6204576

[12] Amrock SM, Zakhar J, Zhou S, Weitzman M. Perception of e-cigarette harm and its correlation with use among U.S. adolescents. Nicotine Tob Res. 2015;17(3):330-336. doi:10.1093/ntr/ntu156 25125321 PMC5479512

[13] Xu Y, Guo Y, Liu K, Liu Z, Wang X. E-cigarette awareness, use, and harm perception among adults: a meta-analysis of observational studies. PLoS One. 2016;11(11):e0165938. doi:10.1371/journal.pone.0165938 27861501 PMC5115669

[14] Sóñora G, Reynales-Shigematsu LM, Barnoya J, Llorente B, Szklo AS, Thrasher JF. Achievements, challenges, priorities and needs to address the current tobacco epidemic in Latin America. Tob Control. 2022;31(2):138-141. doi:10.1136/tobaccocontrol-2021-057007 35241577 PMC8908794

[15] Luhning S, Buljubasich D, Senatore G. Evolution of tobacco control in Latin America: what has been achieved and what is pending. Arch Bronconeumol. 2023;59(8):476-478. doi:10.1016/j.arbres.2023.02.013 36935254

[16] Ceron D, Analuisa P, Valdivieso M D, Mendieta M J. Global youth tobacco survey in Ecuador 2016-2017. Abstract presented at: 17th World Conference on Tobacco or Health; March 7-9, 2018; Cape Town. doi:10.18332/tid/84650

[17] Perucic AM, Sandoval RC, Malik S, Morales-Zamora G. Taxation of novel and emerging nicotine and tobacco products (HTPs, ENDS, and ENNDS) globally and in Latin America. Rev Panam Salud Publica. 2022;46:e175. doi:10.26633/RPSP.2022 36267147 PMC9559335

[18] Instituto Nacional de Estadística y Censos. País atrevido: la nueva cara sociodemográfica del Ecuador. INEC; 2012.

[19] Ministerio de Salud Pública Ecuador. La Ley Orgánica para la Regulación y Control del Tabaco también aplica a cigarrillos electrónicos. Ministerio de Salud Pública; 2018. Accessed January 27, 2024. https://www.salud.gob.ec/la-ley-organica-para-la-regulacion-y-control-del-tabaco-tambien-aplica-a-cigarrillos-electronicos/

[20] Datosmacro. Ecuador - Piramide de población. Accessed January 27, 2024. https://datosmacro.expansion.com/demografia/estructura-poblacion/ecuador

[21] Ortiz-Prado E, Izquierdo-Condoy JS, Fernandez-Naranjo R, et al. A comparative analysis of a self-reported adverse events analysis after receiving one of the available SARS-CoV-2 vaccine schemes in Ecuador. Vaccines (Basel). 2022;10(7):1047. doi:10.3390/vaccines10071047 35891211 PMC9323750

[22] Oyapero A, Erinoso O, Osoba M, Kareem S. Predictors of electronic cigarettes use and its association with mental health in Nigeria: A community survey. Popul Med. 2023;5(December):33. doi:10.18332/popmed/175938

[23] Doherty J, Davison J, McLaughlin M, et al. Prevalence, knowledge and factors associated with e-cigarette use among parents of secondary school children. Public Health Pract (Oxf). 2022;4:100334. doi:10.1016/j.puhip.2022.100334 36389259 PMC9664552

[24] Menezes AMB, Wehrmeister FC, Sardinha LMV, et al. Use of electronic cigarettes and hookah in Brazil: a new and emerging landscape. The Covitel study, 2022. J Bras Pneumol. 2023;49(1):e20220290. doi:10.36416/1806-3756/e20220290 36753212 PMC9970375

[25] Barrera-Núñez DA, López-Olmedo N, Zavala-Arciniega L, Barrientos-Gutiérrez I, Reynales-Shigematsu LM. Consumo de tabaco y uso de cigarro electrónico en adolescentes y adultos mexicanos. Ensanut Continua 2022. Salud Publica Mex. 2023;65:s65-s74. doi:10.21149/14830 38060943

[26] Mirbolouk M, Charkhchi P, Kianoush S, et al. Prevalence and distribution of e-cigarette use among U.S. adults: behavioral risk factor surveillance system, 2016. Ann Intern Med. 2018;169(7):429-438. doi:10.7326/M17-3440 30167658 PMC10534294

[27] Jaber RM, Mirbolouk M, DeFilippis AP, et al. Electronic cigarette use prevalence, associated factors, and pattern by cigarette smoking status in the United States from NHANES (National Health and Nutrition Examination Survey) 2013-2014. J Am Heart Assoc. 2018;7(14):e008178. doi:10.1161/JAHA.117.008178 30007934 PMC6064855

[28] Mahdi HA, Elmorsy SA, Melebari LA, et al. Prevalence and intensity of smoking among healthcare workers and their attitude and behavior towards smoking cessation in the western region of Saudi Arabia: a cross-sectional study. Tob Prev Cessat. 2018;4(August):30. doi:10.18332/tpc/93787 32411856 PMC7205148

[29] Aljandaleh H, Bolze C, El-Khoury Lesueur F, Melchior M, Mary-Krause M. Factors associated with electronic cigarette use among young adults: the French “Trajectoires EpidéMiologiques en POpulation” (TEMPO) Cohort Study. Subst Use Misuse. 2020;55(6):964-972. doi:10.1080/10826084.2020.1717534 31997695

[30] Malt L, Verron T, Cahours X, et al. Perception of the relative harm of electronic cigarettes compared to cigarettes amongst US adults from 2013 to 2016: analysis of the Population Assessment of Tobacco and Health (PATH) study data. Harm Reduct J. 2020;17(1):65. doi:10.1186/s12954-020-00410-2 32948187 PMC7501702

[31] Dwedar I, Ruby D, Mostafa A. A survey exploring knowledge and beliefs about electronic cigarettes between health care providers and the general population in Egypt. Int J Chron Obstruct Pulmon Dis. 2019;14:1943-1950. doi:10.2147/COPD.S214389 32021137 PMC6719839

[32] eClinicalMedicine. E-cigarette use among adolescents: are we doing enough? EClinicalMedicine. 2022;50:101623. doi:10.1016/j.eclinm.2022.101623 36035439 PMC9403831

[33] Unger JB, Falcon A. E-cigarette use among Hispanics: reducing risk or recruiting new tobacco users? Addict Behav. 2022;125:107149. doi:10.1016/j.addbeh.2021.107149 34678711

[34] Martínez-Sánchez JM, Fu M, Martín-Sánchez JC, Ballbè M, Saltó E, Fernández E. Perception of electronic cigarettes in the general population: does their usefulness outweigh their risks? BMJ Open. 2015;5(11):e009218. doi:10.1136/bmjopen-2015-009218 PMC4636602 26534735

[35] Yan XS, D’Ruiz C. Effects of using electronic cigarettes on nicotine delivery and cardiovascular function in comparison with regular cigarettes. Regul Toxicol Pharmacol. 2015;71(1):24-34. doi:10.1016/j.yrtph.2014.11.004 25460033

[36] Huang P, Zheng W, Shi Y, et al. Beliefs and perceptions of electronic cigarettes among medical staff in respiratory departments of Fujian Province, China, in 2021. Tob Induc Dis. 2022;20(December):111. doi:10.18332/tid/156038 36561425 PMC9743794

[37] El ‘vapeo’ un negocio al alza, pese a advertencias. Expreso. March 11, 2023. Accessed January 27, 2024. https://www.expreso.ec/actualidad/economia/vapeo-negocio-alza-pese-advertencias-153463.html

[38] Burgelman S. 41% of teenagers can’t tell the difference between true and fake online health messages. Frontiers Science News. Accessed January 27, 2024. https://blog. frontiersin.org/2022/08/29/psychology-teenagers-health-fake-messages/

