# Supplementary material for: E-cigarette use among Ecuadorian adults: A national cross-sectional study on use rates, perceptions, and associated factors
Source: Tob Induc Dis. 2024 May 31;22:10.18332/tid/187878. doi: 10.18332/tid/187878 (PMC11141133; doi:10.18332/tid/187878)
Supplement: Supplementary file 1 [file TID-22-95-s1.pdf]

Supplementary file 2. Beliefs and Perceptions of Ecuadorian adults about e-cigarettes, according to the prevalence of use, in 2022, n=3047.

**Beliefs and Perceptions**		**EC Use**							
		**Total**	**No**		**Past use**		**Current use**		**P - value**
		**n**	**n**	**%**	**n**	**%**	**n**	**%**	
How much do you know about CE?	Nothing	539	462	85.7%	33	6.1%	44	8.2%	**< 0.001**
	A little bit	1250	984	78.7%	84	6.7%	182	14.6%	
	Moderate knowledge	888	581	65.4%	91	10.2%	216	24.3%	
	A lot	133	69	51.9%	14	10.5%	50	37.6%	
	I'm an expert	34	7	20.6%	3	8.8%	24	70.6%	
Do ECs contain nicotine?	Yes	1797	1200	66.8%	166	9.2%	431	24.0%	**< 0.001**
	No	1047	903	86.2%	59	5.6%	85	8.1%	
Does the EC contain tobacco?	Yes	575	476	82.8%	31	5.4%	68	11.8%	**< 0.001**
	No	2269	1627	71.7%	194	8.6%	448	19.7%	
Can ECs be addictive?	Yes	2509	1868	74.5%	180	7.2%	461	18.4%	**< 0.001**
	No	335	235	70.1%	45	13.4%	55	16.4%	
Does the essence of CE contain chemicals that can cause long-term health damage?	Yes	2623	1949	74.3%	206	7.9%	468	17.8%	0.298
	No	221	154	69.7%	19	8.6%	48	21.7%	
How harmful do you think the use of EC is to health?	Nothing harmful	133	86	64.7%	15	11.3%	32	24.1%	**< 0.001**
	Moderately harmful	1616	1099	68.0%	146	9.0%	371	23.0%	
	Very harmful	1095	918	83.8%	64	5.8%	113	10.3%	
Do you consider that minors (under 18 years old) are suitable to consume EC?	Yes	154	76	49.4%	22	14.3%	56	36.4%	**< 0.001**
	No	2690	2027	75.4%	203	7.5%	460	17.1%	
Do you think EC use has different health effects when compared to tobacco cigarette smoking?	EC is more harmful than tobacco cigarettes	273	178	65.2%	33	12.1%	62	22.7%	**< 0.001**
	EC is less harmful than tobacco cigarettes	1037	679	65.5%	97	9.4%	261	25.2%	
	EC and tobacco cigarettes are equally harmful	1534	1246	81.2%	95	6.2%	193	12.6%	
Do you think EC use is a helpful strategy to quit smoking tobacco cigarettes?	Yes	929	549	59.1%	89	9.6%	291	31.3%	**< 0.001**
	No	1168	976	83.6%	75	6.4%	117	10.0%	
	I don't know	747	578	77.4%	61	8.2%	108	14.5%	
Do you think that the EC should have the same consumption restrictions as tobacco cigarettes?	Yes	2201	1719	78.1%	152	6.9%	330	15.0%	**< 0.001**
	No	643	384	59.7%	73	11.4%	186	28.9%	
Do you consider EC consumption to be fashionable?	Yes	2498	1830	73.3%	200	8.0%	468	18.7%	0.064
	No	346	273	78.9%	25	7.2%	48	13.9%	
	The EC tastes better	504	232	46.0%	82	16.3%	190	37.7%	**< 0.001**

What are the benefits of EC consumption?	EC can be smoked in places where tobacco cigarettes are prohibited	453	296	65.3%	45	9.9%	112	24.7%	
	EC can help me quit smoking tobacco cigarettes	321	193	60.1%	38	11.8%	90	28.0%	
	EC causes less damage to health than tobacco cigarettes	334	255	76.3%	13	3.9%	66	19.8%	
	EC has no health benefit	1232	1127	91.5%	47	3.8%	58	4.7%	
How did you first learn about CE?	Social Media Advertising	887	750	84.6%	47	5.3%	90	10.1%	**< 0.001**
	Advertising on television, radio, or newspaper	208	189	90.9%	6	2.9%	13	6.3%	
	Family or friends	1625	1125	69.2%	147	9.0%	353	21.7%	
	A CE store piqued my interest	124	39	31.5%	25	20.2%	60	48.4%	
Out of 10 of your friends and family, you believe that:	Between 0 and 2 consume EC	1703	1348	79.2%	148	8.7%	207	12.2%	**< 0.001**
	Between 3 and 5 consume EC	819	557	68.0%	56	6.8%	206	25.2%	
	Between 6 and 7 consume EC	207	132	63.8%	17	8.2%	58	28.0%	
	Between 8 and 10 consume EC	115	66	57.4%	4	3.5%	45	39.1%	
Do your parents use e-cigarettes?	Yes	103	39	37.9%	13	12.6%	51	49.5%	**< 0.001**
	No	2741	2064	75.3%	212	7.7%	465	17.0%	
Do you think that people who use EC have an easier time starting to use tobacco cigarettes for the first time?	Yes	2100	1636	77.9%	154	7.3%	310	14.8%	**< 0.001**
	No	744	467	62.8%	71	9.5%	206	27.7%	
Do you think that consuming EC causes those who have quit smoking tobacco cigarettes to do so again?	Yes	1953	1519	77.8%	142	7.3%	292	15.0%	**< 0.001**
	No	891	584	65.5%	83	9.3%	224	25.1%	
Do you consider EC consumption to be a public health problem in Ecuador?	Yes	1885	1467	77.8%	138	7.3%	280	14.9%	**< 0.001**
	No	959	636	66.3%	87	9.1%	236	24.6%	
Do you find it annoying or uncomfortable for people to consume EC near you?	Yes	1340	1208	90.1%	59	4.4%	73	5.4%	**< 0.001**
	No	1504	895	59.5%	166	11.0%	443	29.5%	
Do you think that EC consumption in enclosed public areas should be banned?	Yes	1922	1601	83.3%	128	6.7%	193	10.0%	**< 0.001**
	No	922	502	54.4%	97	10.5%	323	35.0%	

Do you know or have you seen people under 18 years consuming EC?	Yes	2096	1516	72.3%	182	8.7%	398	19.0%	0.003
	No	748	587	78.5%	43	5.7%	118	15.8%	
Do you think that the EC should pay extra taxes as well as tobacco and liquors?	Yes	1962	1646	83.9%	120	6.1%	196	10.0%	**< 0.001**
	No	839	425	50.7%	104	12.4%	310	36.9%	

EC: electronic cigarette. P-values were calculated from the Chi-square test.

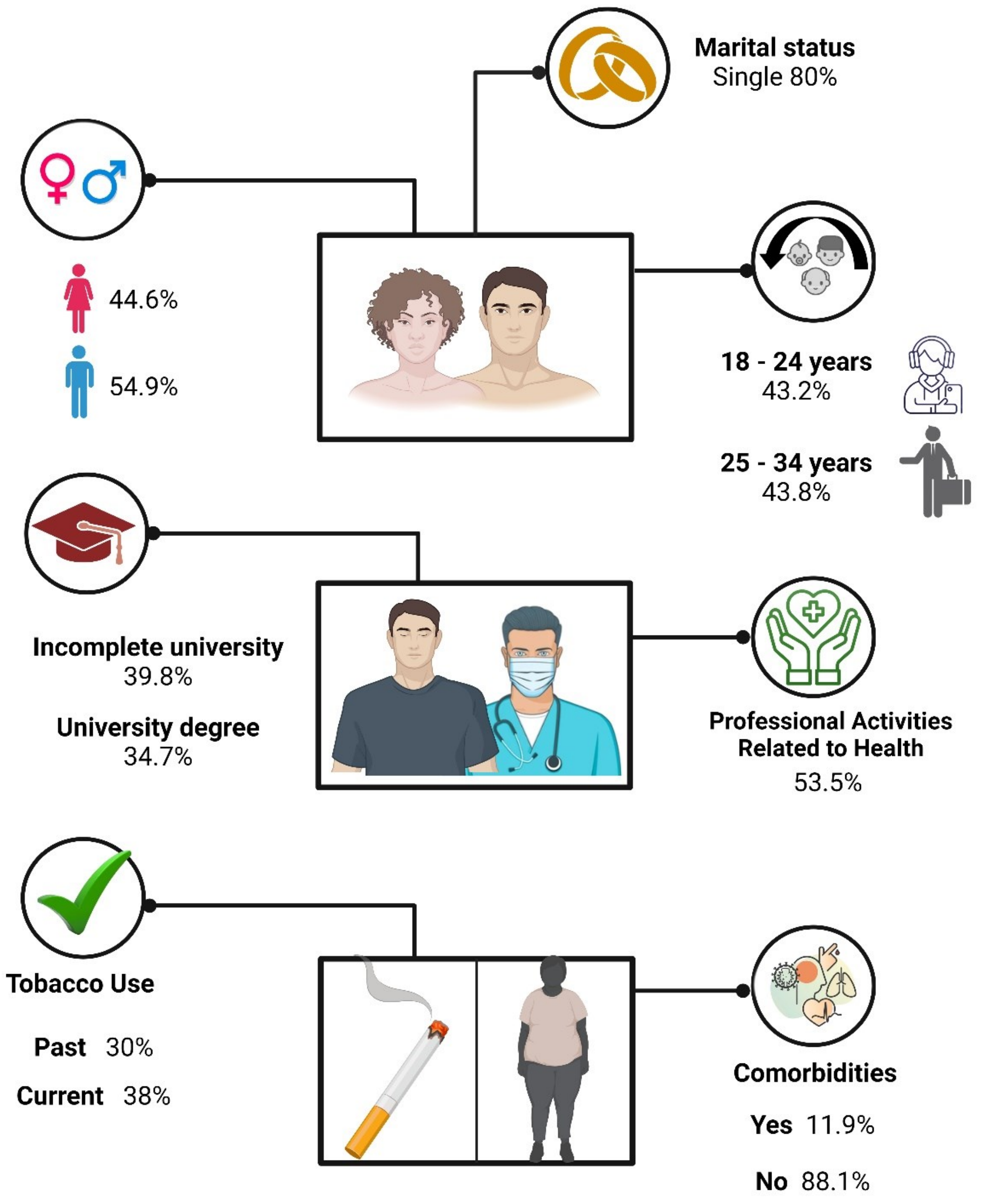

Supplementary Figure 1. Main characteristics of ever users of electronic cigarettes among the adult population of Ecuador, in 2023, n=850.

The Figure shows the percentages of the most frequent characteristics among the group of ever smokers (past + current users) of electronic cigarettes, all percentages were calculated from n=850.

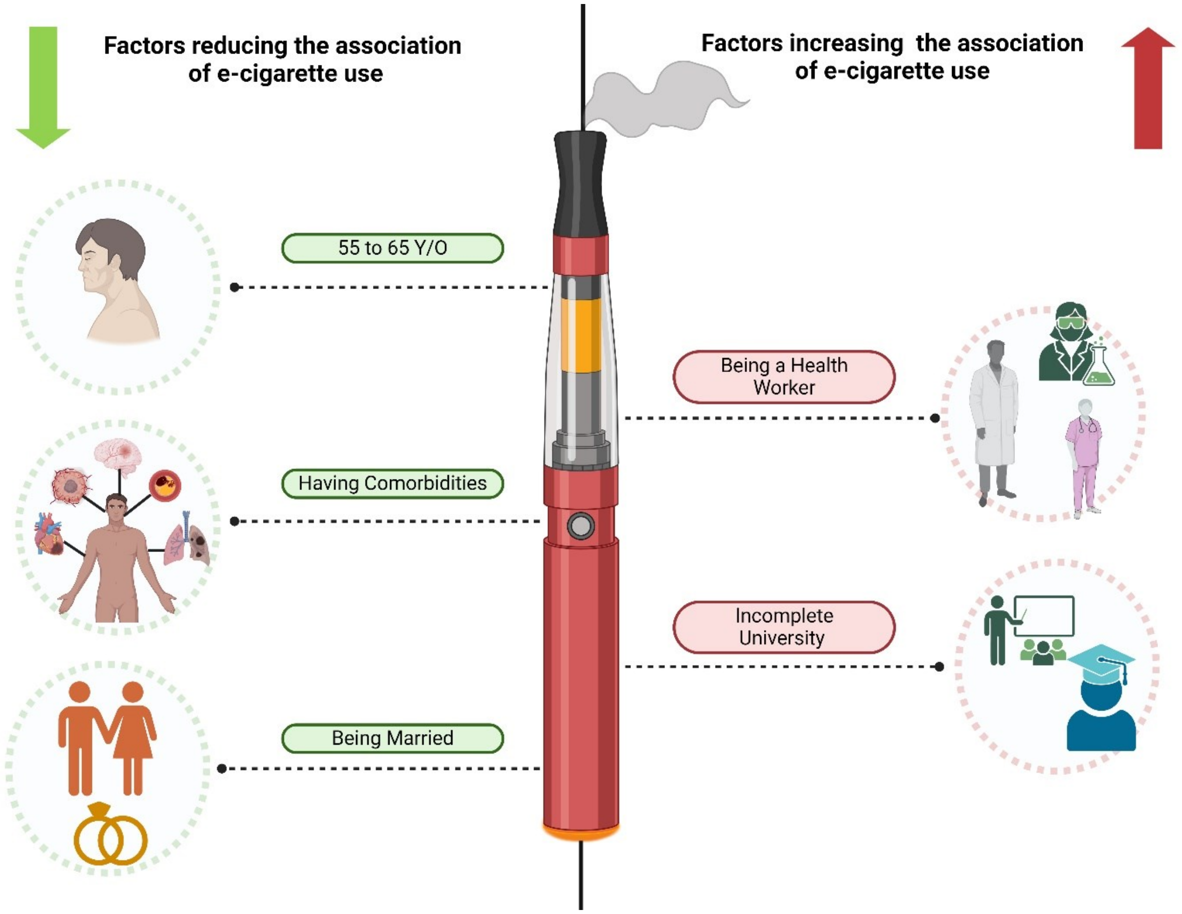

Supplementary Figure 2. Main factors associated with e-cigarette use in the adult population of Ecuador, in 2023, n=3,047.

This figure shows the factors associated with e-cigarette use, the left side shows the factors less associated with e-cigarette use, while the left side shows the factors associated with increased e-cigarette use. All the factors shown in this figure are statistically significant and are the product of an adjusted multivariate analysis.

"Beliefs, knowledge and use of electronic cigarette in adult Ecuadorian population"

Demographics

1. How old are you? (years old)
2. Sex
   - Male
   - Female
   - I'd rather not mention it.
3. What ethnic group do you belong to?
   - Mestizo
   - Indigenous
   - White
   - Afro-descendant
   - Montubio
   - Other
4. Province in which you reside
   - Azuay
   - Bolívar
   - Cañar
   - Carchi
   - Chimborazo
   - Cotopaxi
   - Gold
   - Emeralds
   - Galapagos
   - Guayas
   - Imbabura
   - Loja
   - The Rivers
   - Manabi
   - Morona Santiago
   - Napo
   - Orellana
   - Pastaza
   - Pichincha
   - Saint Helena
   - Santo Domingo de los Tsáchilas
   - Sucumbios
   - Tungurahua
   - Zamora Chinchipe

5. Marital status
    - Single
    - Married
    - De facto union
    - Divorced
    - Widowed
6. What is your academic degree?
    - Incomplete school
    - School completed
    - Secondary (school) Incomplete
    - Secondary (college) Completed
    - Complete University
    - Master/PhD/Specialty
7. Are the professional activities you engage in related to health areas?
    - Yes
    - No

Background

8. Are you diagnosed with any of the following diseases (comorbidities)?
    - Type 1 diabetes
    - Type 2 diabetes
    - Asthma
    - Cancer
    - Hyperthyroidism
    - Hypothyroidism
    - Obesity
    - High blood pressure
    - HIV/AIDS
    - Coagulation disorders
    - Chronic obstructive pulmonary disease
    - No major disease
9. Do you currently smoke cigarettes?
    - Yes
    - No
    - I smoked before

    9. 2 How many years have you been using tobacco cigarettes?
    - *Between 0 and 5 years*
    - *Between 5 and 10 years*
    - *Between 10 and 20 years*
    - *More than 20 years*

    9.3 How many tobacco cigarettes do you consume (on average) during a week?
    - *Between 1 and 10*
    - *Between 11 and 20*
    - *Between 20 and 40*
    - *More than 40*

10. Do you consume or use electronic cigarettes or vapers?
    - *Yes*
    - No
    - I used e-cigarettes before

Beliefs and attitudes

11. How much do you know about e-cigarettes?
    - Nothing at all
    - A little bit
    - Moderate
    - A lot
    - I'm an expert
12. Do e-cigarettes contain nicotine?
    - Yes
    - No
13. Do e-cigarettes contain tobacco?
    - Yes
    - No
14. Can e-cigarettes be addictive?
    - Yes
    - No
15. Does the e-cigarette contain any chemicals that can cause harmful long-term health effects?
    - Yes
    - No
16. How harmful do you think using e-cigarettes is to a person's health?
    - Nothing harmful is harmful
    - Moderately harmful
    - Very harmful
17. Do you think minors can use e-cigarettes?
    - Yes
    - No
18. Do you think e-cigarettes can be less harmful than tobacco cigarettes?
    - The electronic cigarette is more harmful than tobacco cigarettes
    - The electronic cigarette is less harmful than tobacco cigarettes
    - E-cigarettes and tobacco cigarettes are equally harmful
19. Do you think e-cigarettes are a helpful strategy for quitting smoking tobacco cigarettes?
    - Yes
    - No
    - I do not know
20. Do you think e-cigarettes should have the same restrictions as tobacco cigarettes?
    - Yes
    - No
21. Do you think e-cigarettes are all the rage?
    - Yes
    - No

22. For you, which of the following is one of the benefits of e-cigarettes?
    - E-cigarettes taste better
    - E-cigarettes can be smoked in places where tobacco cigarettes are prohibited
    - E-cigarettes can help me quit smoking tobacco cigarettes
    - E-cigarettes cause less health damage than tobacco cigarettes
    - E-cigarettes have no health benefit
23. How did you first learn about e-cigarettes?
    - Social Media Advertising
    - Advertising on television, radio or newspaper
    - Family or friends
    - I was interested in a store
24. Among your friends or family, what percentage use e-cigarettes?
    - Between 0 – 25%
    - Between 26 – 50%
    - Between 51 – 75%
    - Between 76 – 100 %
25. Out of ten of your friends and family, you believe that:
    - Between 0 and 2 consume electronic cigarettes
    - Between 3 and 5 consume electronic cigarettes
    - Between 6 and 7 consume electronic cigarettes
    - Between 8 and 10 consume electronic cigarettes
26. Do your parents use e-cigarettes?
    - Yes
    - No
27. Do you think that people who use electronic cigarettes have an easier time starting to use tobacco cigarettes for the first time?

    -Yes

    -No

28. Do you think that using electronic cigarettes causes those who have quit smoking tobacco cigarettes to do so again?

-Yes

-No

29. Do you think that the use of electronic cigarettes is a public health problem in Ecuador?

-Yes

-No

30. Do you find it annoying or uncomfortable for people to use e-cigarettes near you?

-Yes

-No

31. Do you consider that the consumption of electronic cigarettes in enclosed public areas should be prohibited?

-Yes

-No

32. Do you know or have you seen people under the age of 18 who use e-cigarettes?

-Yes
-No

© 2024 Izquierdo-Condoy J.S. et al.
